# Premature Coronary Artery Disease and Familial Hypercholesterolemia: Need for Early Diagnosis and Cascade Screening in the Indian Population

**DOI:** 10.1155/2012/658526

**Published:** 2011-10-27

**Authors:** N. Setia, I. C. Verma, B. Khan, A. Arora

**Affiliations:** ^1^Center of Medical Genetics, Sir Ganga Ram Hospital, Rajinder Nagar, New Delhi 110060, India; ^2^Atlanta Vascular Research Foundation, Saint Joseph's Translational Research Institute, 13562 Habersham, Northlake, Atlanta, GA 30084, USA; ^3^Hyperlipidemia Prevention Clinic, Department of Cardiology, Sir Ganga Ram Hospital, Rajinder Nagar, New Delhi 110060, India

## Abstract

Cardiovascular disease (CVD) is the leading cause of death in India, accounting for 28% of mortality. The average age of onset of CVD is younger (below 55 years) among Indians than in other populations. This may be due to bad lifestyle, genetic factors, or both. Hypertension, smoking, diabetes, and physical inactivity have been identified as modifiable risk factors for heart disease. Hypercholesterolemia is the most common and treatable cause of heart disease. Genetic factors that lead to hypercholesterolemia have not been fully studied in India. Familial Hypercholesterolemia results from mutations in the LDL receptor, ApoB, PCSK9, and ApoE genes. There is an urgent need to screen subjects with premature CAD and their relatives in India for the presence of FH, identify the mutations that lead to high cholesterol, and carry out cascade screening in the at-risk relatives. Those harbouring mutations in the above genes can be treated to lower the cholesterol levels, prevent early CVD, and avoid death. A programme based on these lines has been initiated in Delhi.

## 1. Introduction

Cardiovascular disease (CVD) is the leading cause of death in India, accounting for 28% of mortality [1]. Deaths from CVD have increased from 2,266,000 in 1990 to 2,669,100 in 2004, which is a 17.8% increase in less than two decades [2]. Prevalence of CVD in the urban Indian population is between 6.5 to 13.2% and in the rural population between 1.6 to 7.4%. The prevalence in the rural areas is growing rapidly possibly due to changing life styles [3].

The average age of onset of CVD is younger (below 55 years) among Indians than in other populations around the world [4]. The case fatality rate for coronary heart disease and other CVDs is higher in India than in the developed countries. This is because effective mechanisms for management and control of CVD are not been fully established and also due to cost of interventions. The heavy burden of CVD in Indians is generally considered to be due to increased incidence of metabolic syndrome and an unhealthy lifestyle. Investigations of the genetic causes for this increased trend have been largely unexplored and the contribution of Familial Hypercholesterolemia (FH) is unknown. 

The Framingham Heart Study (FHS) was started in 1950s when there was growing burden of CVD in the United States. It led to the recognition of risk factors for CVD such as smoking, high blood pressure, an abnormal lipid profile, obesity, diabetes, physical inactivity, and psychosocial factors. This has been shown to be true in all ethnic groups [5]. Asian Indians have been shown to manifest CVD at lower levels of these risk factors, as compared to other populations.

Epidemiological studies have been conducted in India to determine the prevalence and age-specific trends in cardiovascular risk factors among adolescents and young Asian Indians. Major risk factors identified were smoking or tobacco use, obesity, hypertension, dysglycemia, and dyslipidemia. It was observed that the prevalence of multiple cardiovascular risk factors was low in adolescents, but rapid escalation of these risk factors occurred by the age 30–39 years [6].

Arora and Trehan [7] investigated 3020 cases of Indians having Coronary Artery Bypass Graft (CABG) for three or more vessel disease in comparison with those having two or less vessel disease. Risk factors differed in the cases with early as compared with those having late onset of CAD. Cigarette smoking and positive family history of CAD was observed more frequently in those with early onset of the disease, whereas hypertension and diabetes mellitus occurred more frequently in those with late onset of disease.

Goel and colleagues [8] documented that in Indians the risk factors for CAD occur at much lower levels of total cholesterol and low-density lipoprotein cholesterol than other populations. High triglyceride and low high-density lipoprotein levels were observed in the Indian subjects. Younger patients had a more atherogenic lipid profile than the older subgroup with CAD. Smoking and family history of premature CAD were the most common associated risk factors. 

High cholesterol level, the major modifiable risk factor for heart disease, has both an environmental as well as a genetic component. Premature CAD in the Indian population might be due to an unhealthy lifestyle alone or due to genetic factors in combination with an unhealthy lifestyle. The genetic component has been largely ignored in India although it has the highest number of deaths due to heart disease. It would be interesting to determine what fraction of deaths due to CVD are due to genetic factors especially FH. Many countries in the west have introduced MEDPED (Make Early Diagnosis to Prevent Early Death) programs. An index case with FH is identified, and the responsible molecular mutation is determined. Then using a combination of cholesterol levels and mutation studies, the extended family members are screened. The programme has been highly successful in reducing mortality due to CVD in these subjects [9].

Reports are available from the Indian subcontinent of cases with Homozygous FH that have been identified on dermatological and cardiological features [10–12]. In all these cases, the patients had a very characteristic distribution of planar xanthomas in interdigital spaces of fingers along with tendinous and some tuberous xanthomas at other places. It was observed that most of these patients had a family history of xanthomas, premature deaths, and deranged plasma cholesterol levels in siblings or the parents. The treatment options available are lipid lowering drugs, LDL apheresis, and liver transplantation [13–15]. Genetic characterization in such families would enable early diagnosis and provision of therapy at a younger age. Guidelines have been developed in UK for diagnosis and treatment of patients of FH and cascade testing among family members [16]. 

Hypercholesterolemia (monogenic and multifactorial) affects 1 in 20 subjects in the general population. On the other hand, frequency of FH is 1 in 500 for heterozygotes. Homozygotes are rare with a frequency of 1 in a million. FH is characterized by isolated elevation of plasma low-density lipoprotein cholesterol and is associated with high risk of premature cardiovascular disease. The affected families can be segregated in three clear groups on the basis of their plasma LDL cholesterol concentrations: presumed homozygotes with levels four times higher than in normal individuals (>800 mg/dL), heterozygotes with levels two times higher than the normal (200–800 mg/dL), and unaffected individuals (100 mg/dL). This is due to the gene dosage effect [17]. It is estimated that there are about 10,000,000 people with FH worldwide [18]. Of these affected subjects, less than 10% are treated with LDL-lowering drugs. If undiagnosed and untreated, the cumulative risk of CAD by age 60 years is more than 60% among men and 30% among women with heterozygous FH [19]. This risk ratio could be much higher in Indians due to the early occurrence of CAD.

## 2. The Genetic Equation in FH

Familial Hypercholesterolemia results from defects in hepatic uptake and degradation of LDL via the LDL-receptor pathway. The high-density lipoprotein cholesterol levels are seen to be in the normal range or low in patients with FH. The defects are generally caused by loss of function mutations in the LDL receptor gene, by mutations in gene encoding apolipoprotein B, or by gain of function mutations in PCSK9 gene.

### 2.1. LDL Receptor Gene

 In 1985, Brown and Goldstein [20] discovered the LDL receptor, a cell surface glycoprotein that binds apolipoprotein B on the LDL particle as a part of the process of receptor mediated endocytosis. The human LDLR receptor cDNA and gene were cloned and characterized in 1984 and 1985, respectively [21]. LDL receptor gene is located on the distal short arm of chromosome 19, in the region p13.1-p13.3. LDLR gene spans 45 kb and comprises of 18 exons and 17 introns. The cellular defects of LDL-receptor function are classified into five groups: defects in ligand binding, transport, internalization, recycling, and null alleles which are the receptor negative mutations. The first molecular characterization of LDL receptor mutation was reported in 1985 by Lehrman et al. [22]. More than 900 mutations in the LDLR have been reported since its discovery [23]. Various mutations found include point mutations, large rearrangements, premature stop codons, single amino acid substitutions and insertions [24–26]. Not much work has been done in the field of molecular genetic characterization of FH in the Indian population. A study on Indians in South Africa found mutations in exons 3, 4, 9, and 14 of LDLR gene. The mutations majorly occurred in the CpG rich regions of the gene making it a mutational hotspot in South African Indians [27]. Unrelated families had different mutations. Ashavaid et al. in a study in Mumbai identified four previously known mutations and two novel insertion mutations in LDLR gene in Indian subjects [28].

### 2.2. ApoB

 Some patients with hypercholesterolemia have no defect in LDL receptor, but have defective clearance of LDL due to mutations in ApoB gene. In such patients, cholesterol concentrations in plasma can vary from those found in heterozygous FH to only modest hypercholesterolemia [29]. The defect lies in inability of the LDL to bind to LDLR receptor due to defective ApoB, the protein moiety of LDL [30]. The defect is known as Familial Defective Apolipoprotein B-100 (FDB). Mutations are found only in the LDL binding domain of ApoB, which consists of exon 26 and 27 of ApoB gene. In a study conducted in 40 subjects with clinical features of FH in India, none of the patients or controls showed a mutation in exon 26 of ApoB. This suggests that common mutations in ApoB are not associated with hypercholesterolemia among Indians [31]. However the possibility of other unknown mutations and polymorphisms at the ApoB locus cannot be ruled out.

### 2.3. PCSK9

 Another locus causing autosomal dominant hypercholesterolemia was identified to be a gene on chromosome 1-Proprotein Convertase Subtilisin/Kexin type IX (PCSK9) [32]. This gene encodes for a protein of 694 amino acids belonging to a family of proprotein convertase subtilase. PCSK9 is secreted by hepatocytes and appears to downregulate the density of functional LDL receptors in hepatocytes by promoting endosomal degradation rather than recycling of the receptor on the surface [33]. Hypercholesterolemia is caused by gain-of-function mutations in the PCSK9 gene [34]. Since its discovery, various missense mutations in PCSK9 have been shown to cosegregate with severe hypercholesterolemia in many families in several countries. It was observed that ApoE genotypes exerted their respective effects on LDL cholesterol in an additive manner to that of the PCSK9 variants [35].

### 2.4. ApoE

 It is a key protein in the modulation of metabolism of highly atherogenic ApoB containing lipoproteins. ApoE is polymorphic in nature and three common alleles, that is, *ε*4, *ε*3, and *ε*2 code for three major isoforms, that is, ApoE4, E3, and E2. Six different ApoE phenotypes: E3/3, E4/4, E2/2, E4/3, E3/2, and E4/2 occur in the general population with varying frequencies. It has been estimated that ApoE polymorphisms may account for 2–16% of the variability of LDL cholesterol levels [36]. In Asian Indians, these allele frequencies observed were 0.031–0.094 for *ε*2, 0.803–0.968 for *ε*3, and 0.000–0.133 for *ε*4 [37]. Frequency of ApoE *ε*3 allele was found to be high (0.913) in people of north India [38]. In another study on Asian Indians, individuals with at least one *ε*4 allele were considered to be at risk to develop premature myocardial infarction, independent of other conventional risk factors [39].

## 3. Conclusions

Special efforts are required to identify individuals with FH in India as they are at high risk of premature coronary heart disease. The condition is seriously underdiagnosed and the diagnosis is often made too late, restricting the benefits of the treatments available. Since this condition is genetically determined, families must become the focus of attention. Cascade testing can identify more individuals with FH who will benefit from early treatment, result in a near-normal life expectancy. The innovative use of DNA testing allied with cholesterol assay will help to ensure that children, young people, and adults with this condition are identified and offered timely advice and treatment. A cost-effective method is being developed to screen for mutations in families with cases having premature CAD. This will help to establish a program in India similar to the MEDPED program. With this initiative many lives will be saved from premature CAD and early death.
